# Case Report: Ultrasound-guided percutaneous splenic biopsy as a lifesaving diagnostic modality in B-cell lymphoma presenting as hemophagocytic lymphohistiocytosis: a report of two cases

**DOI:** 10.3389/fonc.2026.1885572

**Published:** 2026-06-12

**Authors:** Zhuohang Zou, Yang Dai, Xushu Zhong, Jun Wang, Ailin Zhao, Yijun Wu, Ting Niu

**Affiliations:** 1 Department of Hematology, Institute of Hematology, and Center for High Altitude Medicine, West China Hospital, Sichuan University, Chengdu, Sichuan, China; 2 West China School of Medicine, Sichuan University, Chengdu, Sichuan, China; 3 National Facility for Translational Medicine (Sichuan), West China Hospital, Sichuan University, Chengdu, Sichuan, China; 4 State Key Laboratory of Biotherapy, Collaborative Innovation Center of Biotherapy, West China Hospital, Sichuan University, Chengdu, Sichuan, China; 5 Department of Medical Ultrasound, West China Hospital, Sichuan University, Chengdu, Sichuan, China

**Keywords:** diagnostic dilemma, hemophagocytic lymphohistiocytosis, lymphoma, splenic lymphoma, ultrasound-guided percutaneous splenic biopsy

## Abstract

**Background:**

Hemophagocytic lymphohistiocytosis (HLH) as the initial manifestation of B-cell lymphoma poses significant diagnostic challenges, particularly when conventional tissue biopsy is contraindicated due to severe cytopenias and coagulopathy.

**Case description:**

We report two cases of B-cell lymphoma initially presenting with HLH, fulfilling HLH-2004 diagnostic criteria. Given the predominant splenic involvement and high bleeding risk, ultrasound-guided percutaneous splenic biopsy was performed, yielding a definitive histopathological diagnosis and guiding subsequent targeted therapy.

**Conclusion:**

Ultrasound-guided percutaneous splenic biopsy may serve as a viable diagnostic option in carefully selected patients with lymphoma-associated HLH and predominant splenic involvement, particularly when conventional approaches are inadequate or contraindicated. Its application requires thorough multidisciplinary risk-benefit assessment.

## Background

Hemophagocytic lymphohistiocytosis (HLH) is a rare, life-threatening hyperinflammatory syndrome characterized by uncontrolled immune activation, cytokine storm, and multi-organ dysfunction (1). Its clinical presentation is often nonspecific, with persistent fever, cytopenias, hepatosplenomegaly, and marked hyperferritinemia serving as hallmark features (2, 3). HLH is broadly classified into primary (familial) forms, driven by germline mutations in genes regulating lymphocyte cytotoxicity, and secondary (acquired) forms, which arise in the context of diverse triggering conditions (4, 5). The etiological spectrum of secondary HLH is remarkably broad, encompassing infections (particularly Epstein-Barr virus), autoimmune and autoinflammatory disorders, malignancies, and immunosuppressive therapies (6, 7). Among these, malignancy-associated HLH (M-HLH) represents a substantial and increasingly recognized proportion, with hematological neoplasms accounting for the vast majority of cases. Lymphomas, in particular, constitute the single largest category of underlying malignancies in adults with secondary HLH (8). The incidence of HLH in patients with lymphoma has been reported to range from 1% to over 10% in certain aggressive subtypes, and the syndrome portends a dismal prognosis when the underlying malignancy remains untreated (9–11).

However, achieving a definitive pathological diagnosis of lymphoma in patients presenting with HLH poses formidable challenges. The profound cytopenias, coagulopathy, and hemodynamic instability characteristic of active HLH render many patients unfit for invasive diagnostic procedures (12). Bone marrow examination, while routinely performed, frequently fails to demonstrate unequivocal lymphomatous infiltration, particularly in cases where the disease is primarily extramedullary. Moreover, bone marrow examination alone is often insufficient to determine the specific subtype of lymphoma, which may compromise subsequent precision therapy (13, 14). When splenic involvement is suspected based on imaging findings—such as splenomegaly with heterogeneous or hypermetabolic lesions—splenectomy has traditionally been considered the gold standard for obtaining diagnostic tissue (15). Yet in the context of concomitant HLH, splenectomy carries prohibitively high perioperative morbidity and mortality risks, including catastrophic hemorrhage, infection, and exacerbation of the cytokine storm. This creates a diagnostic paradox: the very condition necessitating urgent histopathological confirmation simultaneously precludes the conventional surgical approach to obtaining it.

Ultrasound-guided percutaneous splenic biopsy, though historically regarded with caution due to concerns regarding hemorrhagic complications, has evolved into a safer procedure with advances in imaging guidance, needle technology, and operator expertise (16). In carefully selected patients, it offers a minimally invasive alternative that may circumvent the risks of splenectomy while still yielding adequate tissue for comprehensive pathological analysis. Nevertheless, data specifically evaluating the feasibility, safety, and diagnostic yield of splenic biopsy in patients with HLH remain scarce, and the procedure is not widely utilized in this vulnerable population.

Herein, we report two cases of B-cell lymphoma presenting with HLH in which ultrasound-guided percutaneous splenic biopsy provided the critical diagnostic modality. In both instances, extensive workup including bone marrow examination failed to identify the definitive underlying malignancy, and the patients’ clinical status precluded splenectomy. Splenic biopsy established the diagnosis of B-cell lymphoma, enabling immediate initiation of lymphoma-directed therapy and resulting in clinically meaningful responses.

## Case description

### Case 1

A 56-year-old male patient was admitted to our medical center on September 2, 2025 due to recurrent fever for 3 months. Three months ago, the patient developed recurrent fever. Relevant examinations at a local hospital revealed cytopenia, elevated ferritin and soluble interleukin-2 receptor (sCD25), and hemophagocytosis, leading to a suspicion of HLH. ED regimen (Etoposide plus Dexamethasone, specific dosages unknown) was given and screening for underlying diseases associated with HLH was also arranged. Whole-body PET-CT revealed diffuse splenomegaly with hypermetabolism, hypermetabolic bone marrow, multiple hypermetabolic subcutaneous soft tissue nodules, a hypermetabolic nodule at the right acromion, and bilateral adrenal thickening with increased uptake, raising suspicion for tumor involvement. Bone marrow aspiration demonstrated unclassifiable cells, suggestive of a neoplasm. Metagenomic next-generation sequencing (mNGS) of pathogenic microorganisms identified co-infection with Klebsiella pneumoniae and Pneumocystis jirovecii. Therefore, anti-infective therapy was also administered. With these treatments, the patient’s symptoms improved and he was discharged.

**Figure 1:**
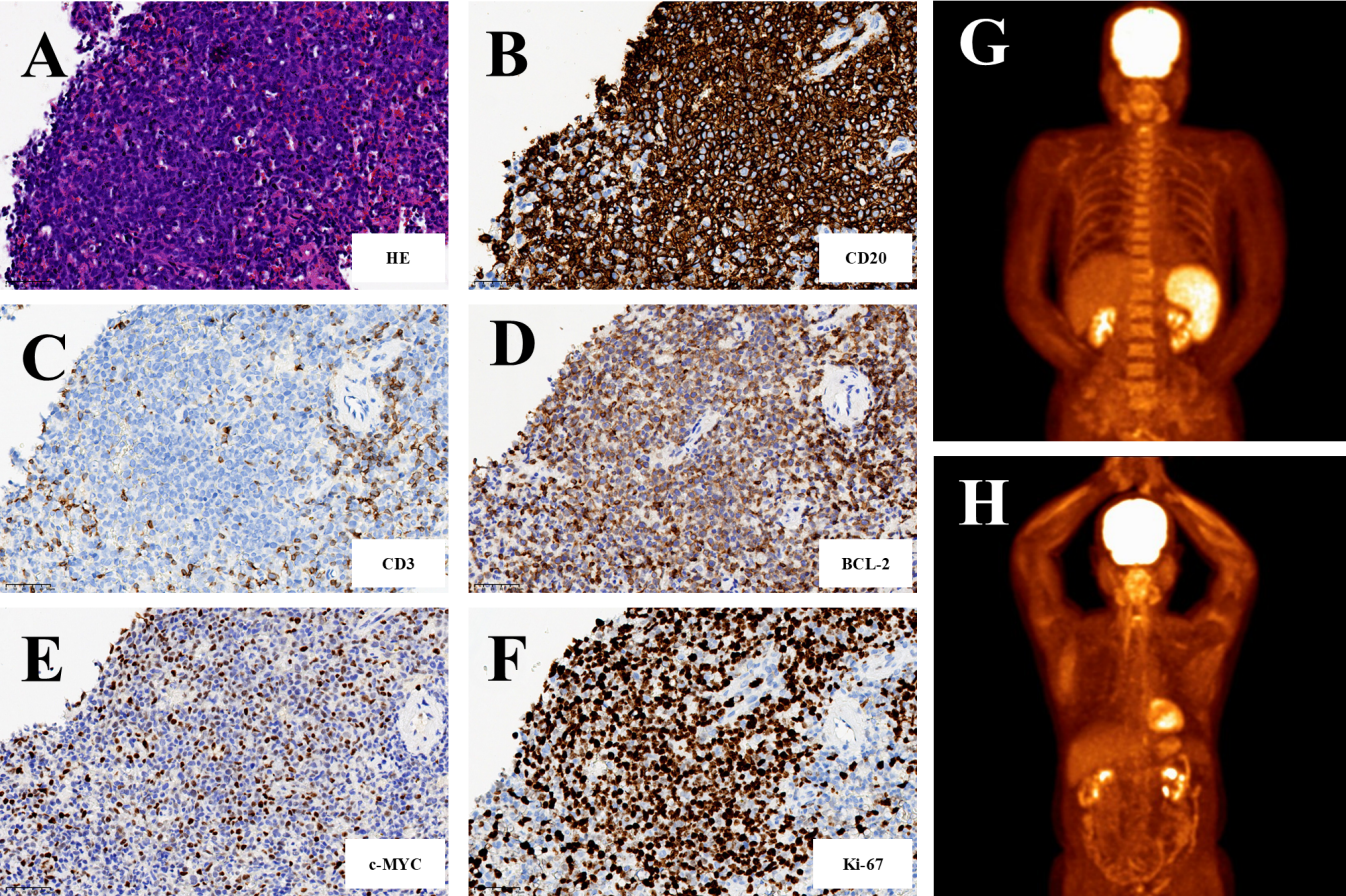
Pathological and PET/CT images of Case 1. **(A–F)** Immunohistochemical staining of spleen biopsy specimens (original magnification ×40): **(A)** H&E staining showing dense, diffuse infiltration of monotonous, hyperchromatic tumour cells; **(B)** CD20 showing diffuse strong positivity in tumour cells; **(C)** CD3 negative in tumour cells; **(D)** BCL-2 demonstrating strong expression (>80%); **(E)** c-MYC showing partial positivity (~40%); **(F)** Ki-67 demonstrating a high proliferation index. **(G)** Baseline whole-body ¹^8^F-FDG PET/CT showing intense hypermetabolic activity in the spleen. **(H)** Post-treatment ¹^8^F-FDG PET/CT demonstrating a marked reduction in metabolic activity.

Ten days later, fever recurred with a peak temperature of 39 °C, and the patient presented to our institute. Laboratory tests revealed the following: hemoglobin 63 g/L (130–175 g/L); platelet count 65×10^9^/L (100–300×10^9^/L); white blood cell count (WBC) 6.68×10^9^/L (3.5–9.5×10^9^/L); creatinine 118 μmol/L (68–108 μmol/L); triglycerides 3.33 mmol/L (0.29–1.70 mmol/L); lactate dehydrogenase (LDH) 226 U/L (120–250 U/L); ferritin 2759 ng/mL (30–400 ng/mL); sCD25–14290 U/mL (223.0–710.0 U/mL); C-reactive protein (CRP) 63.3 mg/L (0.0–6.0 mg/L). Based on persistent fever, splenomegaly, bicytopenia, hyperglyceridemia, elevated ferritin, and elevated sCD25, the diagnostic criteria for HLH-2004 were met (6 of 8 criteria fulfilled); therefore, a diagnosis of HLH was established. The ED regimen was administered (etoposide 100 mg twice weekly and dexamethasone 10 mg once daily), while active investigation for underlying diseases associated with HLH was undertaken. A 42-gene hemophagocytic syndrome mutation panel revealed no pathogenic variants. EBV-DNA was below the lower limit of detection. In conjunction with the examination data from the outside hospital, a neoplastic disease—particularly lymphoma—was highly suspected. Therefore, whole-body PET/CT was arranged again, which demonstrated enlarged spleen with diffuse increased 18F-FDG uptake; maximum SUV 6.88. CT showed no definite abnormal density lesions within the splenic parenchyma; diffuse increased glucose metabolism in axial skeleton and proximal appendicular bone marrow; no other significant abnormalities were detected (**Figure 1**).

**Figure 2:**
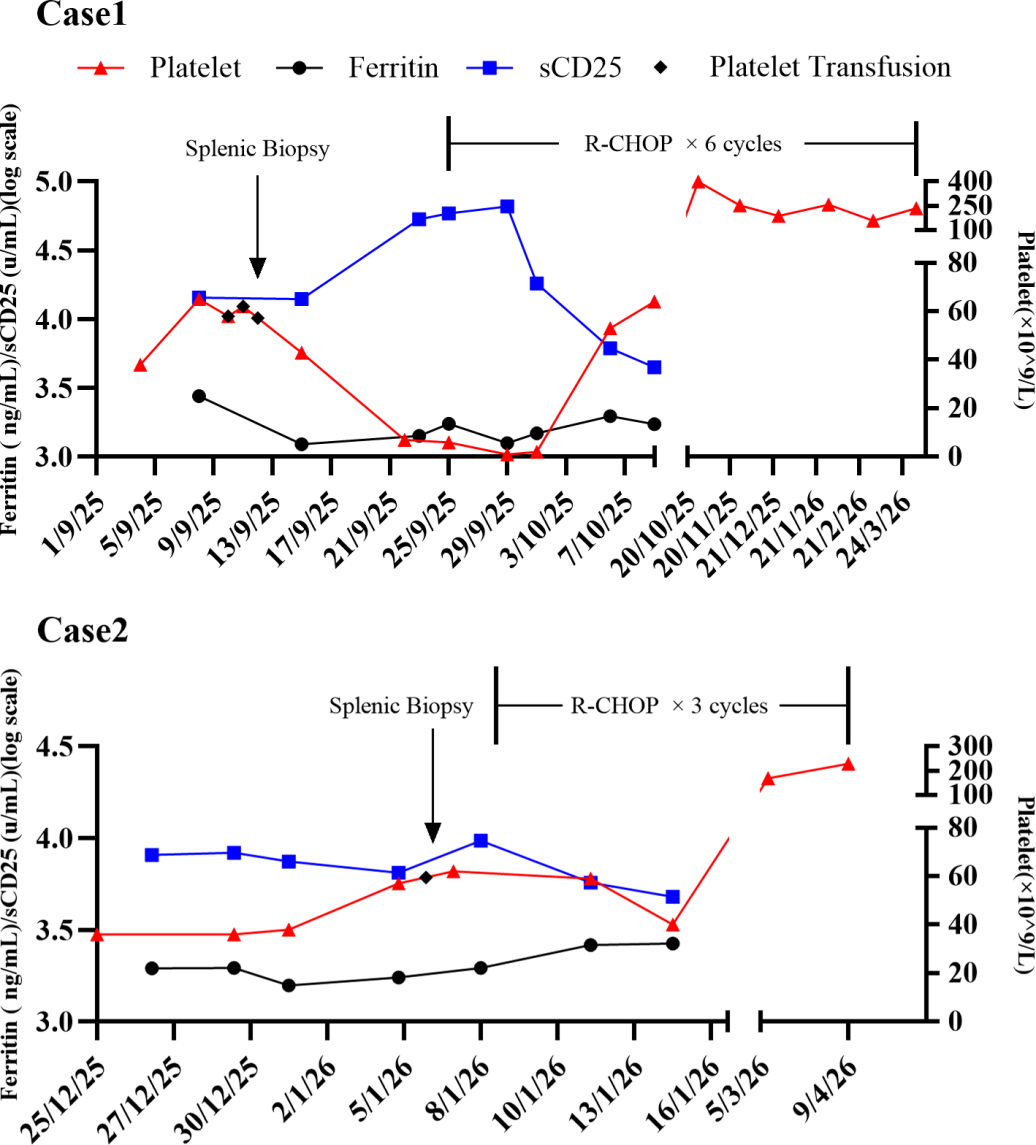
Treatment course and temporal evolution of laboratory parameters. Ferritin (black) and sCD25 (blue) levels are shown on a logarithmic scale (left y-axis), while platelet counts (red, right y-axis) are presented over time. Platelet transfusions are indicated by black squares with corresponding units administered.

Bone marrow smear, flow cytometry and biopsy revealed no obvious abnormalities. In light of these results, splenic lymphoma was highly suspected. Traditionally, suspected splenic lymphoma is diagnosed by splenectomy with pathological biopsy. However, in this patient, given the concurrent HLH, poor general condition, and thrombocytopenia, splenectomy may not be tolerable. Moreover, the PET/CT did not reveal any other accessible sites for biopsy. Therefore, after multidisciplinary discussion, a decision was made to perform ultrasound-guided percutaneous core needle biopsy of the spleen. On September 12, 2025, after transfusion of three units of platelets, the patient’s platelet count increased to 62×10^9^/L upon re-examination. Ultrasound-guided splenic biopsy was performed as follows: the puncture site was sterilised and draped, and the ultrasound probe was covered with a sterile protective sheath. Local anaesthesia was administered using 2% lidocaine. Under real-time ultrasound guidance, an 18G biopsy needle was advanced through a 17G coaxial needle into the splenic parenchyma, and tissue specimens were obtained under continuous sonographic monitoring. Post-procedure needle tract bleeding was observed; gelatin sponge was deployed along the tract to achieve haemostasis, with successful cessation of bleeding. Following 30 minutes of compression at the puncture site, repeat ultrasound examination confirmed cessation of bleeding and demonstrated no active haemorrhage in the needle tract region or significant anechoic free fluid in the peritoneal cavity. Upon return, the patient was placed under cardiac monitoring for 24 hours. Vital signs remained stable, and the patient denied abdominal pain or other symptoms. Due to financial constraints, the patient requested discharge to await pathological results as an outpatient. One week later, splenic tissue pathology was available. Immunohistochemical staining showed that the tumor cells were CD20(+), CD79a(+), CD3(−), CD10(−), BCL6(+), MUM1(+), CD23(−), PD-1(−), CD30(−), CD5(−), cyclin D1(−), TdT(−), BCL2(+, >80%), c-MYC(+, ~40%), p53(+, 10%–20%), Ki-67(+, >80%). *In situ* hybridization for EBER1/2 was negative. Gene rearrangement analysis (PCR with GENESCAN analysis) identified clonal amplification peaks for immunoglobulin heavy chain (IgH) and kappa light chain (IgK), confirming B-cell clonality. These pathological findings confirm a diagnosis of diffuse large B-cell lymphoma (WHO classification; invasive, high proliferative activity; **Figure 1**). According to the Hans algorithm, the tumor was classified as having a non-germinal center B-cell (non-GCB) origin. The tumor exhibited dual expression of BCL2 and c-MYC. Fluorescence *in situ* hybridization (FISH) showed no rearrangements of the BCL2, BCL6, or MYC genes. The patient was readmitted and commenced on the CR-CHOP regimen (chidamide 20 mg twice weekly, rituximab 600 mg on day 1, cyclophosphamide 1200 mg day 2, epirubicin 100 mg day 2, vindesine 4 mg day 2, prednisone 50 mg twice daily d2–6). Fifteen days after the first cycle of R-CHOP, repeat laboratory evaluation showed platelet count increased to 64×10^9^/L. The patient was discharged and received three consecutive cycles of R-CHOP. Notably, platelet counts remained stable throughout the inter-treatment periods, with no recurrence of cytopenias suggestive of HLH relapse. Following four cycles of R-CHOP, restaging PET/CT demonstrated complete metabolic response (CMR) consistent with complete remission (CR). The patient is currently undergoing ongoing chemotherapy (**Figure 2**).

### Case 2

A 70-year-old female patient presented on December 25, 2025, with fever and cough persisting for more than two weeks. Two weeks prior to presentation, she developed fever (maximum temperature 41 °C) accompanied by dizziness, fatigue, and dry cough. The patient presented to a local hospital, where laboratory examinations showed hemoglobin 87 g/L, platelet count 54×10^9^/L, and ferritin 2579.8 ng/mL. Bone marrow smear demonstrated hemophagocytic cells and 2% of unclassifiable cells, lymphoma origin considered. Flow cytometric analysis showed clonal plasma cells in bone marrow accounting for approximately 5.91% (with strong CD19 and CD45 expression and partial CD20 expression). Then she was transferred to our hospital. Physical examination revealed splenomegaly with the spleen palpable 4 cm below the left costal margin; the liver was not palpable. Laboratory findings were as follows: hemoglobin 71 g/L; platelet count 34×10^9^/L; WBC 3.64×10^9^/L; triglycerides 2.01 mmol/L; ferritin 1964.00 ng/mL; sCD25–8337 U/mL; LDH 206 IU/L; procalcitonin (PCT) 0.144 ng/mL.

**Figure 3:**
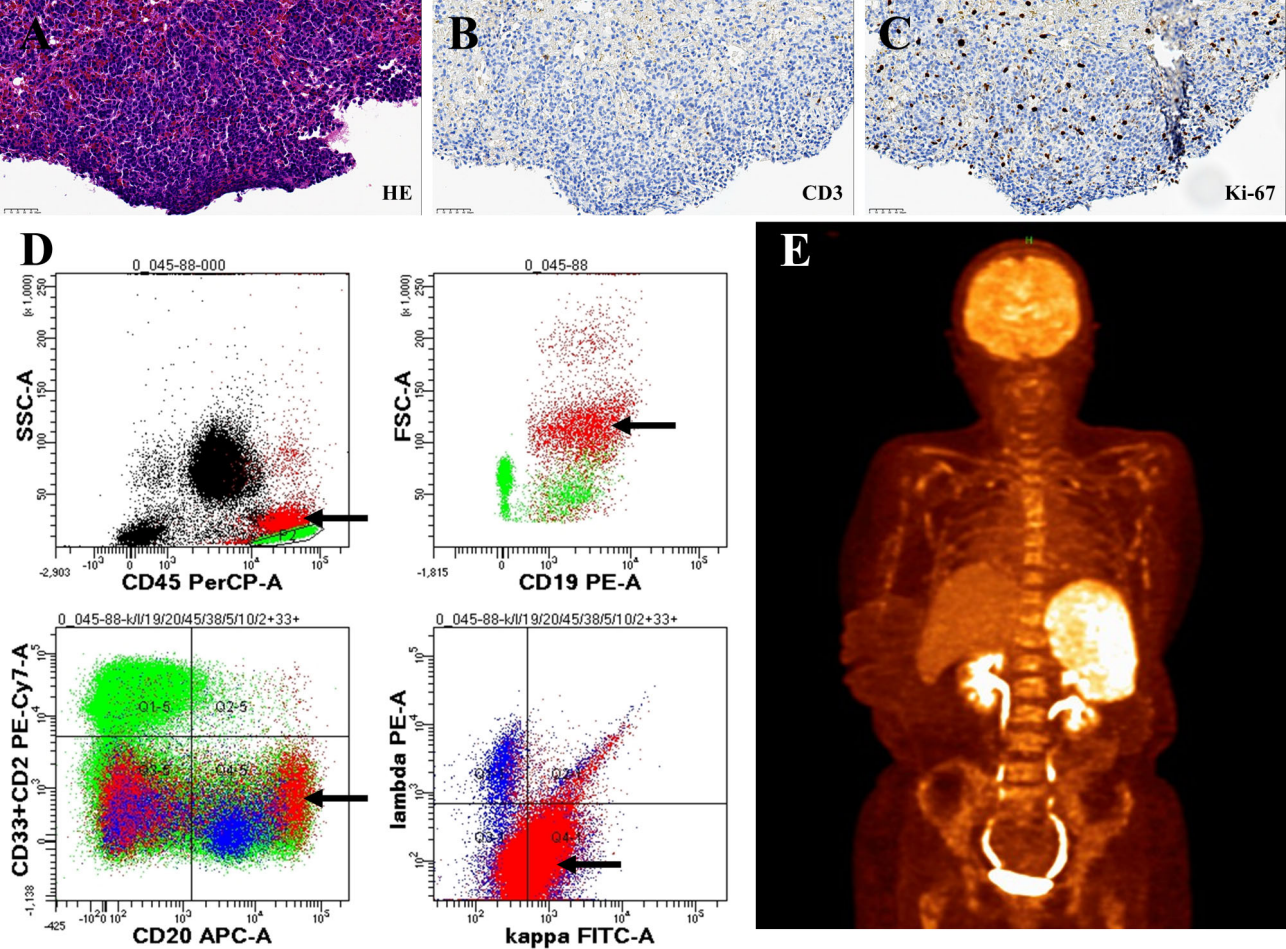
Flow cytometric, Pathological and baseline PET/CT images of Case 2. **(A–C)** Immunohistochemical staining of spleen biopsy specimens (original magnification ×40): **(A)** H&E staining showing dense, diffuse infiltration of monotonous, hyperchromatic tumour cells; **(B)** CD3 negative in tumour cells; **(C)** Ki-67 demonstrating a low proliferation index. **(D)** Flow cytometric analysis identifying a clonal B-cell population constituting 10% of nucleated cells, with increased forward scatter consistent with larger cell size, CD20 positivity and restricted kappa light chain expression. **(E)** Baseline whole-body ¹^8^F-FDG PET/CT showing intense hypermetabolic activity in the spleen.

The patient met the HLH-2004 diagnostic criteria with six of eight fulfilled: fever, splenomegaly, bicytopenia, hyperferritinemia, elevated sCD25, and hemophagocytosis in the bone marrow. Therefore, ED regimen (etoposide 80 mg twice weekly plus dexamethasone 10 mg once daily) was initiated. Concurrent workup for the underlying etiology of HLH was performed. Abdominal CT scan revealed: Splenomegaly; wedge-shaped slightly hypodense lesion in posterior spleen. Small amount of perisplenic fluid; PET/CT demonstrated splenomegaly with increased glucose metabolism, suggestive of hematologic malignancy; splenic infarction suspected; diffusely increased glucose metabolism of the bone marrow (**Figure 3**). Bone marrow smear examination revealed 6.5% abnormal lymphoid cells with morphological atypia and evidence of hemophagocytosis. Flow cytometric analysis identified a clonal B-cell population accounting for 10% of nucleated cells, characterized by increased forward scatter (FSC) consistent with larger cell size. The immunophenotype was as follows: positive for CD19, CD20, CD38, CD22 (bright), and FMC7 (partial); dim expression of CD5; restricted κ light chain expression; and negative for CD10, CD23, CD103, CD200, and λ light chain. These findings were consistent with a clonal B-cell lymphoproliferative disorder showing a CD5(dim)CD10(−) immunophenotype and increased cell size. Bone marrow biopsy revealed scattered and small focal infiltrates of lymphocytes and plasma cells. Immunohistochemical staining revealed lymphocytes positive for CD20 (approximately 50%–60%) and focally positive for CD3, consistent with bone marrow involvement by B-cell lymphoma. Next-generation sequencing with a 42-gene panel for hemophagocytic lymphohistiocytosis was performed. Two variants of uncertain significance (VUS) were identified: a missense variant in UNC13D and a missense variant in STXBP2. No pathogenic or likely pathogenic variants were detected.

The aforementioned findings were highly suggestive of lymphoma as the underlying etiology of HLH. However, bone marrow examination was insufficient to establish a definitive diagnosis or specific subtype of lymphoma. Imaging studies indicated that the pathological process was predominantly localized to the spleen. Given the patient’s advanced age, concomitant thrombocytopenia, and coagulopathy, a multidisciplinary discussion was conducted. After careful risk–benefit assessment, ultrasound-guided percutaneous splenic biopsy was ultimately deemed appropriate.

On January 6, 2026, after transfusion of 2 units of platelets, the platelet count was rechecked and found to be 57×10^9^/L. Coagulation test results were normal. Ultrasound-guided percutaneous splenic biopsy was then performed using the same procedural protocol as in the previous case. Immunohistochemical staining of the splenic biopsy specimen showed the following immunophenotype: CD20(−), CD79a(+), CD3(−), CD19(+), CD5(dim/+), CD23(−), CD10(−), BCL6(+, scant), BCL2(+), cyclin D1(−), CD43(−), CD30(−), IgG4(−), IgD(−), MUM1(+), CD138(+, partial), CD56(−), PCK(−), PD-1(−). CD21 staining demonstrated absence of a follicular dendritic cell (FDC) meshwork. The Ki-67 proliferation index was low (3%–5%). *In situ* hybridization (ISH) for EBER1/2 was negative. Clonal immunoglobulin heavy chain (IgH) and kappa light chain (IgK) gene rearrangements were detected, and no clonal T-cell receptor gamma (TCRG) rearrangement was identified. Pathological evaluation confirmed a B-cell lymphoid neoplasm; the favored diagnosis was an indolent B-cell lymphoma with marked plasmacytic differentiation (weakly CD5-positive; **Figure 3**). Although the pathological diagnosis favored an indolent B-cell lymphoma, the overall clinical picture was more consistent with aggressive B-cell lymphoma, possibly representing transformation from an indolent precursor. This interpretation was supported by (1): markedly elevated splenic SUVmax on PET/CT, indicative of high metabolic activity (2); increased FSC on bone marrow flow cytometry, reflecting larger cell volume; and (3) concomitant HLH, a recognized complication of high-grade lymphoproliferative disorders. Once lymphoma was confirmed as the underlying etiology of HLH, etoposide plus dexamethasone was discontinued, and the patient was transitioned to lymphoma-directed therapy. Given the patient’s advanced age and poor performance status, dose-reduced R-CHOP chemotherapy (rituximab 500 mg on day 1 + cyclophosphamide 600 mg day 2 + epirubicin 50 mg day 2+ vindesine 3 mg day 2 + prednisone 30 mg bid day 2-5) was initiated. Despite CD20 negativity on histopathology, flow cytometry of bone marrow identified an abnormal lymphoid population with CD20 positivity; therefore, rituximab was administered. Following the first cycle of R-CHOP, the patient developed myelosuppression complicated by febrile neutropenia (peak temperature 38.9 °C). Empirical broad-spectrum antimicrobial therapy with meropenem was initiated. The fever resolved within 48 hours. Repeat complete blood count demonstrated: WBC 2.46×10^9^/L, Hb 61 g/L and PLT 40×10^9^/L. Eltrombopag was administered to support platelet recovery, and the patient was subsequently discharged. Three weeks after discharge, the platelet count recovered to 64×10^9^/L, and eltrombopag was discontinued. The patient subsequently continued with chemotherapy cycles and platelet counts remained within normal range, with no evidence of HLH relapse (**Figure 2**).

## Discussion

In both patients, lymphoma-associated HLH manifested as severe cytopenias, coagulopathy, and predominant splenic involvement, rendering conventional diagnostic approaches inadequate or contraindicated. Although HLH itself can involve the spleen and produce characteristic pathological changes related to uncontrolled immune activation, including red pulp histiocytic expansion, haemophagocytosis, and reactive inflammatory infiltrates (17, 18), the markedly increased FDG uptake predominantly localized to the spleen on PET/CT in these two patients raised concern for underlying lymphomatous involvement. Therefore, definitive tissue diagnosis remains essential when lymphoma-associated HLH is suspected. Ultrasound-guided percutaneous splenic biopsy circumvented these obstacles, providing a definitive B-cell lymphoma diagnosis without major haemorrhagic complications and enabling prompt initiation of curative-intent therapy. These observations underscore several clinically important considerations: the diagnostic limitations of bone marrow examination in this setting, the substantial constraints on splenectomy imposed by critical illness, the safety and feasibility of image-guided splenic tissue acquisition in high-risk patients, and the profound therapeutic impact of histological confirmation. However, while these findings support the diagnosis of HLH, they do not reliably distinguish reactive haemophagocytic activity from an underlying malignant trigger. Consequently, in patients with suspected lymphoma-associated HLH, splenic tissue evaluation may provide not only evidence of haemophagocytosis but also critical diagnostic information regarding occult lymphomatous involvement. The diagnostic criteria for HLH in both patients are shown in **Table 1**.

**Table 1:** HLH diagnostic criteria fulfilled by two patients.

HLH diagnostic criteria fulfilled by two patients			
HLH-2004 Diagnostic criteria		Case 1	Case 2
Fever (> 38.5 °C)		Yes (Maximum 39.0 °C; fever persisted > 1 week)	Yes (Maximum 41.0 °C; fever persisted > 1 week)
Splenomegaly		Yes	Yes
Cytopenias (Affecting ≥ 2 lineages)	Hemoglobin (<90 g/L)	Yes (63 g/L)	Yes (71 g/L)
	Platelet (<100×10^9/L)	Yes (65×10^9/L)	Yes (34×10^9/L)
	Neutrophils (<1.0×10^9/L)	No	No
Hypertriglyceridemia (Triglycerides ≥ 3.0 mmol/L or ≤ 265 mg/dL)		Yes (Triglycerides 3.33 mmol/L)	No
or Hypofibrinogenemia (Fibrinogen ≤ 1.5 g/L)		No	No
Ferritin (≥ 500 ng/mL)		Yes (2,759 ng/mL)	Yes (1,964 ng/mL)
sCD25 (≥ 2,400 U/mL)		Yes (14290 U/mL)	Yes (8,337 U/mL)
Hemophagocytosis (bone marrow, spleen, or lymph nodes)		No	Yes (Bone marrow)
Decreased or absent natural killer (NK) cell activity		NA	NA

### Diagnostic limitations of bone marrow examination in lymphoma-associated HLH

Bone marrow evaluation is central to HLH diagnosis, demonstrating haemophagocytosis and detecting underlying malignancy. However, its capacity to definitively establish lymphoma as the triggering aetiology is frequently compromised. The marrow microenvironment in lymphoma-associated HLH presents a challenging diagnostic landscape. Profound cytopenias, coagulopathy, and hypercytokinaemia create hypocellularity and reactive histiocytic hyperplasia that obscure neoplastic infiltrates. Haemophagocytic activity itself may dominate the morphological picture, diverting attention from subtle malignant populations (19, 20). In one of our cases, marrow examination demonstrated haemophagocytosis yet failed to provide definitive lymphoma classification. Flow cytometry offers enhanced sensitivity for clonal detection but cannot preserve tissue architecture, precluding definitive subtyping of aggressive lymphomas such as DLBCL (21, 22). It also cannot distinguish between the aggressive clone driving HLH and incidental low-grade disease—a distinction with profound therapeutic implications (23). Patchy marrow infiltration compounds sampling error, as standard trephine biopsies may miss focal lesions. In lymphoma-associated HLH, malignant cells often preferentially localise to extramedullary sites—particularly spleen, liver, and lymph nodes—whilst minimally involving marrow. Our cases exemplify this pattern: the diagnostic breakthrough required directed splenic tissue acquisition.

Importantly, marked splenomegaly in HLH is not specific for lymphoma involvement and may occur secondary to immune activation, histiocytic proliferation, and haemophagocytic activity. Therefore, splenic enlargement alone should not be interpreted as evidence of malignant infiltration. In the present cases, however, PET/CT demonstrated predominant splenic FDG hypermetabolism, a pattern that raised strong suspicion for underlying lymphomatous splenic involvement rather than reactive splenic changes associated with HLH. Combined with nondiagnostic bone marrow findings, these imaging features supported the need for directed splenic tissue acquisition to establish a definitive diagnosis and guide subsequent therapy.These limitations mandate that bone marrow examination be regarded as initial but insufficient in lymphoma-associated HLH. When marrow fails to yield definitive classification—particularly with predominant visceral involvement on imaging—alternative tissue acquisition strategies must be pursued without delay.

### Limitations of splenectomy in HLH patients

Splenectomy has historically been considered the diagnostic gold standard for suspected splenic lymphoma, providing ample tissue for histological classification, immunophenotyping, and molecular analysis (15). However, this approach is substantially limited in patients presenting with HLH. The syndrome is characterised by profound systemic inflammation, multi-organ dysfunction, and severe cytopenias, rendering patients physiologically unfit for major abdominal surgery. Thrombocytopenia, often refractory to platelet transfusion due to peripheral consumption, splenic sequestration, and marrow suppression, poses an unacceptable haemorrhagic risk (24). Concurrent coagulopathy, driven by hepatic dysfunction, hypofibrinogenaemia, and disseminated intravascular coagulation, further compounds perioperative morbidity and mortality. Additionally, the post-splenectomy immunocompromised state, coupled with functional asplenia, may exacerbate the already heightened infection risk in these critically ill, often neutropenic patients (25). Prolonged operative time and anaesthetic exposure in haemodynamically unstable patients may precipitate further clinical deterioration. Consequently, splenectomy is frequently contraindicated or pragmatically deferred until clinical stabilisation, by which time the therapeutic window for curative intervention may have irrevocably closed. These substantial constraints necessitate the development and adoption of less invasive, bedside-compatible alternatives for tissue diagnosis in this exceptionally vulnerable population.

### Feasibility of ultrasound-guided splenic biopsy in HLH patients

Percutaneous splenic biopsy has historically been regarded with caution due to the organ’s vascularity and friable parenchyma, with conventional teaching emphasising the risk of catastrophic haemorrhage. However, accumulating evidence supports the safety of ultrasound-guided approaches when performed with appropriate precautions (16). In HLH patients, where splenectomy is contraindicated and marrow examination nondiagnostic, percutaneous splenic biopsy emerges as a viable alternative. Real-time ultrasound guidance enables precise needle placement, avoiding major vessels and areas of necrosis, whilst permitting immediate detection of post-procedural complications. Two techniques are available for percutaneous splenic biopsy: core needle biopsy and fine-needle aspiration biopsy. Our center adopts core needle biopsy with semi-automatic spring-loaded biopsy needles of 18-gauge. Core needles have a wider bore than fine-needle aspiration devices, allowing acquisition of larger tissue samples. Published data indicate a 12% complication rate for percutaneous splenic biopsies with 14-gauge needles and rare complication rates with 22-gauge aspiration needles (26). To date, we have found no studies comparing the diagnostic sensitivity of these two biopsy modalities for splenic lymphoma. Pre-procedural optimisation with platelet transfusion to thresholds generally exceeding 50×10^9^/L and correction of coagulopathy are essential prerequisites, as mandated by our institutional protocol. The procedure should be performed by experienced interventional radiologists with immediate access to cross-matched blood products and surgical backup.

In both cases reported herein, ultrasound-guided percutaneous splenic biopsy was successfully performed without major haemorrhagic complications, yielding diagnostic tissue that established the diagnosis of lymphoma. These observations align with emerging literature supporting the safety of image-guided splenic biopsy in thrombocytopenic patients when meticulous haemostatic protocols are observed (27–29). The diagnostic yield in lymphomatous splenic involvement is high, as the disease typically diffusely infiltrates the parenchyma rather than forming focal lesions that might be missed by needle sampling (30). The establishment of definitive histological diagnosis carries profound therapeutic implications in lymphoma-associated HLH. Without tissue confirmation, patients frequently receive empiric HLH-directed therapy—etoposide plus corticosteroids—which, whilst temporarily suppressing hyperinflammation, fails to address the underlying malignant driver (31, 32). In both cases, splenic biopsy confirmed a diagnosis of aggressive large B-cell lymphoma, allowing immediate initiation of R-CHOP chemotherapy. This transition from empiric HLH management to lymphoma-directed therapy resulted in rapid resolution of HLH activity and sustained cytopenia recovery. These outcomes underscore that tissue diagnosis is not merely an academic exercise but a critical determinant of survival, directly enabling the therapeutic pivot upon which prognosis depends.

### Broader implications, limitations, and future directions

These cases support an imaging-directed, multidisciplinary diagnostic algorithm for lymphoma-associated HLH. When bone marrow examination is nondiagnostic and superficial lymphadenopathy absent, functional imaging should guide tissue acquisition: predominant hepatic involvement favours transjugular liver biopsy, whilst isolated splenomegaly with intense FDG avidity indicates percutaneous splenic biopsy. This anatomical stratification maximises diagnostic yield whilst minimising procedural risk in critically ill patients. Limitations include inherent selection bias, small sample size, and the requirement for experienced interventional radiology and immediate surgical standby. Splenic biopsy in profoundly thrombocytopenic patients demands rigorous haemostatic optimisation that may not be universally feasible. Furthermore, the optimal platelet threshold and coagulation parameters for safe splenic biopsy in this population remain undefined.

Future efforts should prospectively validate this imaging-directed approach across multicentre cohorts. Ultimately, early diagnostic precision through minimally invasive, risk-adapted strategies represents the critical first step toward improving dismal outcomes in this challenging population.

In conclusion, lymphoma-associated HLH presents a formidable diagnostic challenge when conventional tissue acquisition strategies are precluded by critical illness. The cases reported herein demonstrate that ultrasound-guided percutaneous splenic biopsy, performed with appropriate haemostatic support and multidisciplinary coordination, can safely provide definitive histological diagnosis in patients with severe thrombocytopenia and coagulopathy. This minimally invasive approach enabled prompt initiation of curative-directed lymphoma therapy and clinically meaningful responses. These findings support the integration of imaging-directed splenic biopsy into the diagnostic algorithm for HLH patients with predominant splenic involvement and nondiagnostic bone marrow examination. Future prospective studies should validate the safety and efficacy of this strategy across broader cohorts and explore its combination with emerging liquid biopsy technologies to further refine diagnostic precision in this vulnerable population.

## Data Availability

The original contributions presented in the study are included in the article/supplementary material. Further inquiries can be directed to the corresponding author/s.
